# The ERM protein Moesin is essential for neuronal morphogenesis and long-term memory in *Drosophila*

**DOI:** 10.1186/s13041-017-0322-y

**Published:** 2017-08-29

**Authors:** Patrick S. Freymuth, Helen L. Fitzsimons

**Affiliations:** Institute of Fundamental Sciences, Massey University, Palmerston North, New Zealand

**Keywords:** Moesin, Ezrin, Radixin, ERM, Cytoskeleton, Actin, *Drosophila*, Memory, Neuron, Courtship, Synaptic plasticity

## Abstract

Moesin is a cytoskeletal adaptor protein that plays an important role in modification of the actin cytoskeleton. Rearrangement of the actin cytoskeleton drives both neuronal morphogenesis and the structural changes in neurons that are required for long-term memory formation. Moesin has been identified as a candidate memory gene in *Drosophila*, however, whether it is required for memory formation has not been evaluated. Here, we investigate the role of Moesin in neuronal morphogenesis and in short- and long-term memory formation in the courtship suppression assay, a model of associative memory. We found that both knockdown and overexpression of Moesin led to defects in axon growth and guidance as well as dendritic arborization. Moreover, reduction of Moesin expression or expression of a constitutively active phosphomimetic in the adult *Drosophila* brain had no effect on short term memory, but prevented long-term memory formation, an effect that was independent of its role in development. These results indicate a critical role for *Moesin* in both neuronal morphogenesis and long-term memory formation.

**Electronic supplementary material:**

The online version of this article (10.1186/s13041-017-0322-y) contains supplementary material, which is available to authorized users.

## Introduction

Moesin belongs to the ERM (Ezrin/Radixin/Moesin) family of proteins, a group of adaptor molecules that are essential organizers of specialized membrane domains, which have been implicated in various fundamental physiological processes including the regulation of cell shape, motility and signaling. For review, see [1, 2]. ERMs maintain the structural stability of the cell cortex by linking transmembrane proteins to the actin cytoskeleton via an N-terminal FERM domain and a C-terminal actin-binding domain [1, 3]. Regulation of ERM activity is facilitated through head to tail folding in which an intramolecular association between the N- and C-terminal domains results in a “closed”, inactive conformation. Phosphorylation of a conserved threonine residue in the C-terminal actin-binding domain relieves this intermolecular association resulting in an “open”, active conformation and the unmasking of ligand-binding sites [3].

ERMs play a critical role in regulation of the cytoskeletal rearrangements that lead to changes in cell shape [4–10]. Activation of ERMs occurs via phosphorylation of threonine 558 through activation of kinases such as Rho-kinase [11]. Constitutive activation of RhoA, which activates Rho-kinase, induces the formation of microvilli-like structures at the apical membrane of fibroblasts, and this is enhanced on co-expression of T559D, a constitutively active phosphomimetic of Moesin. However co-expression of the non-phosphorylatable mutant T559A inhibits formation of the RhoA-induced microvilli-like structures, indicating that phosphorylation of Moesin is essential for this growth process [11]. Similarly, in epithelial cells, the constitutively active form of Ezrin, T567D, associates with the actin-rich plasma membrane and induces the growth of actin-rich projections, but the inactive form, T567A, does not [12]. In *Drosophila*, Moesin is required for photoreceptor morphogenesis where it facilitates normal assembly of the apical membrane skeleton of the rhabdomere. When expressed during photoreceptor morphogenesis, the constitutively active mutant T559D concentrates at the apical membrane, resulting in a profusion of irregular microvilli [7].

In neurons, rearrangements in the actin cytoskeleton underpin neuronal morphogenesis and synaptic plasticity [13–16]. A key process driving neuronal morphogenesis is the guidance of the growing axons toward synaptic targets [17] and the dynamic activity of the growth cone is characterized by persistent extension and withdrawal of actin-rich membrane protrusions, which bear membrane receptors that detect extrinsic guidance cues [18, 19]. Moesin and Radixin have been identified as prominent components of axonal growth cones of cultured rat hippocampal pyramidal neurons, with the double suppression of their expression leading to disorganization of F-actin and defects in morphology and motility [20]. Phosphorylation of Moesin is required for nerve growth factor-mediated outgrowth of PC12 cell neurites [21], and exposure of hippocampal neurons to glutamate induces activation of Moesin and is associated with an increase in the number of active synaptic boutons, the presynaptic axon terminals that contact dendritic spines to form a synapse [22]. This increase is diminished by Moesin knockdown as well as impairment of ERM phosphorylation, indicating that ERMs may be involved in the synaptic response to activity [22].

Rearrangement of the actin cytoskeleton also drives the structural changes that occur in dendritic spines, which are believed to underlie memory formation and maintenance [15, 16, 23, 24]. Progestogen and estrogen both induce cytoskeletal remodeling in cortical neurons, which is coincident with phosphorylation of Moesin via a signaling cascade involving RhoA and the Rho-associated kinase, ROCK-2 [25, 26]. These hormones are critical modulators of neuronal morphology and function and have been demonstrated to play a critical role not only in brain development but also learning and memory [27–29]. Activation of this pathway is associated with increased dendritic spine density and a redistribution of Moesin to membrane sites where spines are formed, while shRNA-mediated silencing of Moesin abrogates this spine growth [25, 26]. These data together indicate that Moesin regulates activity-dependent cytoskeletal rearrangements and dendritic spine growth, suggesting a potential role in the structural changes that are thought to underpin memory formation. Indeed, Moesin has been identified as a candidate memory gene through DNA microarray analysis of the *Drosophila* transcriptional response following training in the olfactory conditioning paradigm, which found that Moesin transcription was induced after spaced relative to massed training [30]. Since spaced but not massed training leads to the formation of protein synthesis-dependent long-term memory [31], this transcriptional response suggests that Moesin may be involved in long-term memory formation.

Despite this accumulating evidence, there have been no studies to date examining whether ERMs play a specific role in memory. As *Drosophila* has a single ERM orthologue *Moesin*, sharing 58% amino acid identity with its human counterpart, analyses are not hindered by the functional redundancy of the ERMs that has been previously observed in vertebrate studies [32]. This advantage, combined with *Drosophila*’s amenability to genetic manipulation and the well-established memory assays that have been developed, provides an informative means for investigation of the role of ERMs in learning and memory. Here, we found that knockdown of Moesin as well as its constitutive activation in the adult *Drosophila* brain prevented long-term memory formation, indicating an essential role in this process, which was independent of its role in development. Moreover, knockdown of Moesin impaired dendritic arborization, whereas constitutive activation appeared to increase the intensity of dendritic protrusions, suggesting Moesin may promote memory formation through facilitation of cytoskeletal rearrangements at synapses.

## Results

### Characterization of Moesin expression in the *Drosophila* brain

**Fig. 1:**
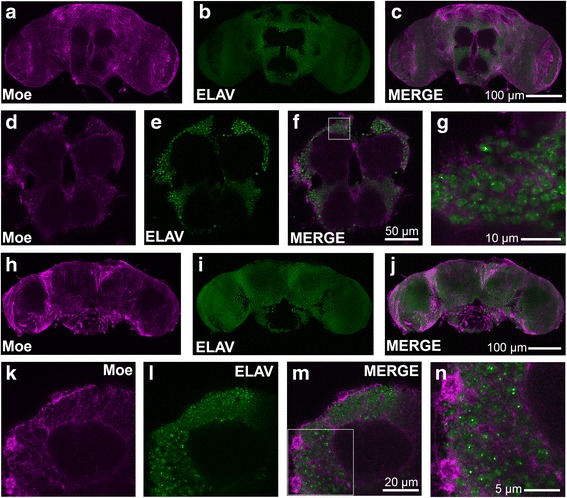
Expression and subcellular localization of Moesin in the brain. Whole mount brains were subjected to immunohistochemistry with anti-Moesin (magenta) and anti-ELAV (green) antibodies. **a**–**c**. frontal confocal projection through the brain illustrating widespread Moesin expression. **d**–**f**. One micron optical slice through the central lobes of the brain illustrating non-nuclear Moesin expression, appearing as a cytoplasmic halo around the ELAV stained nuclei. **g**. Magnification of area surrounded by the white square in **f**. **h**–**j**. Posterior confocal projection through the brain. **k**–**m**. One micron optical slice through the calyx illustrating non-nuclear Moesin expression in Kenyon cells. **n**. Magnification of area surrounded by the white square in **m**

We first sought to characterize the expression pattern of Moesin in the *Drosophila* brain, which has not been previously examined. Immunohistochemical staining of whole mount brains revealed widespread expression of Moesin throughout all regions of the brain (Fig. 1a, h). The subcellular distribution of Moesin was non-nuclear and predominantly cytoplasmic, as observed by the lack of colocalization with ELAV, a marker of neuronal nuclei (Fig. 1b–j) and the Moesin-positive cytoplasmic haloes surrounding the ELAV-positive nuclei (Fig. 1d–g). In the mushroom body, a region of the brain critical for memory formation and recall [33, 34], Moesin was not observed in the lobes (axons) of the Kenyon cells, the intrinsic neurons of the mushroom body (Fig. 1a; see Additional file 1: Figure S1B to visualize the location of the lobes in the brain), however magnification of the cell bodies of the Kenyon cells revealed cytoplasmically localized Moesin (Fig. 1m, n).

**Fig. 2:**
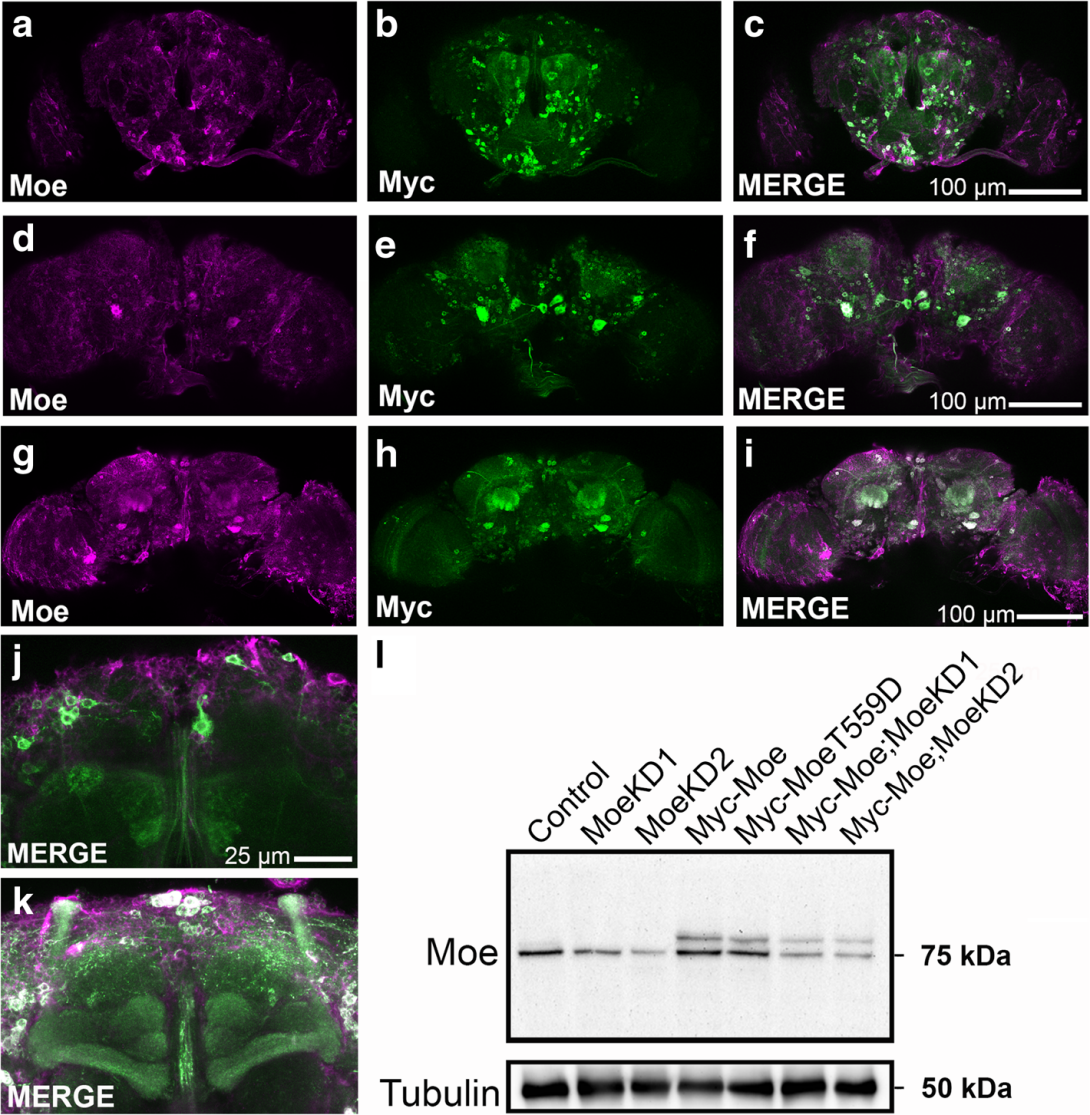
Characterisation of *Moesin* knockdown and overexpression. **a**–**k**. Immunohistochemistry with anti-Moesin (magenta) and anti-Myc (green) antibodies on whole mount brains. In all brains, elav-GAL4-mediated expression was restricted to the adult brain with the TARGET system. **a**–**c**. frontal confocal projection through a brain expressing Myc-Moe. **d**–**f**. Posterior confocal projection through a brain expressing Myc-Moe. **g**–**i**. Posterior confocal projection through a brain expressing Myc-MoeT559D. **j**, **k**. Confocal projection through a mushroom body expressing Myc-Moe (**j**) and Myc-Moe T559D (**k**). **l**. Western blot shows the expression of Moesin in head lysates of flies in which *Moesin* is overexpression or knocked down. The wild-type strain *w1118* was also crossed to elav-GAL4 as a control genotype. Blots were probed with anti-Moesin to detect endogenous Moesin as well as Myc-Moe and Myc-MoeT559D. Anti-α-tubulin antibody was used as a loading control

In order to investigate the importance of Moesin in neuronal development as well as in learning and memory in adult flies, we genetically manipulated the level of Moesin expression in the brain via the UAS/GAL4 system combined with the pan-neuronal elav-GAL4 driver. The resulting expression patterns of the transgenic constructs were then examined via immunohistochemistry on whole mount brains. As overexpression of Moesin could potentially affect neuronal development, expression was induced in adulthood via the TARGET system, which utilizes a temperature-sensitive repressor of GAL4 transcription, GAL80ts. Flies were raised at a GAL80 permissive temperature (19 °C) until two days after eclosion, at which time expression was induced at the restrictive temperature (30 °C) for 48 h as expected. The expression pattern of a Myc-tagged wild-type Moesin transgene (Myc-Moe) was very similar to that of endogenous Moesin (Fig. 2a–f). Expression of the phosphomimetic Myc-MoeT559D, a constitutively active mutant of Moesin [7] also resulted in robust expression throughout the brain (Fig. 2g–i). However, unlike Myc-Moe, Myc-MoeT559D was targeted to the mushroom body lobes (compare Fig. 2j and k) and also displayed a stronger presence in the calyx (compare Fig. 2e and h). This pattern mimics that of Lifeact, a GFP-tagged actin-binding peptide (Additional file 1: Figure S1) [35], indicating that on activation, Moesin redistributes from the cytoplasm to actin-rich regions of the neuron.

Appropriate overexpression and knockdown of Moesin was also confirmed via western blot. A specific band of approximately 75 kDa, the estimated molecular weight of *Drosophila* Moesin, was detected in whole-cell lysates of wild-type *Drosophila* heads (Fig. 2l), whereas expression of each RNAi resulted in a reduced signal, confirming that each targeted Moesin. Expression of Myc-Moe was detected as a slightly higher molecular weight band in addition to endogenous Moesin, as was Myc-MoeT559D. In the two strains co-expressing Myc-Moe and a Moesin RNAi construct, the levels of both endogenous Moesin and Myc-Moe proteins are reduced, which is expected as both endogenous Moesin and the Myc-Moe constructs contain the mRNA sequences targeted by RNAi.

### Altered Moesin expression disrupts mushroom body development

**Fig. 3:**
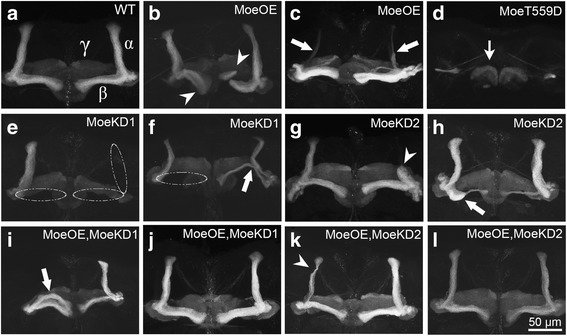
Altered Moesin expression disrupts mushroom body development. **a**–**h**. Immunohistochemistry with anti-FasII antibody on whole mount brains reveals mushroom body defects resulting from elav-GAL4 driven expression of UAS-Moe constructs. All images are frontal confocal projections through the mushroom body region of the brain. Scale bar = 50 μm. **a**. Wild-type mushroom body. α, β and γ lobes of the mushroom body are labeled in white. **b**. Misoriented β lobes (arrowheads) in a fly expressing Myc-Moe. **c**. Thin, reduced, α lobe projections in a fly expressing Moe-KD2. **d**. Complete disruption of mushroom body development in a fly expressing Myc-MoeT559D, with thin, distorted γ lobes (arrow). **e**. Missing α and β lobes (dashed lines) in a fly expressing Moe-KD1. **f**. Missing β lobe (dashed line) and α/β branching defect (arrow) in a fly expressing Moe-KD1. **g**. Axon stalling defect characterized by a partially formed α lobe (arrowhead) in a fly expressing Moe-KD2. **h**. β lobe outgrowth defect (arrow) in a fly expressing Moe-KD2. **i**. Misdirected α lobe (arrow) in a fly co-expressing Myc-Moe and Moe-KD2. **j**. Rescue of mushroom body development through coexpression of Myc-Moe with Moe-KD1. **k**. Thin α lobe (arrowhead) in a fly co-expressing Myc-Moe and Moe-KD1. **l**. Rescue of mushroom body development through coexpression of Myc-Moe with Moe-KD2

In light of the demonstrated role of Moesin in neuronal morphogenesis [7], we first investigated the impact of Moesin knockdown and overexpression on mushroom body development. Immunohistochemical staining for the neuronal marker FasII strongly labels the α and β lobes of the mushroom body and weakly labels the γ lobe enabling the visualization of mushroom body lobes [36]. In the wild-type brain, the axons of the α and β neurons each project from the cell bodies in a bundled fiber termed the peduncle, and then bifurcate to form the vertical and horizontal α and β lobes (Fig. 3a). Both elav-GAL4 driven overexpression of Myc-Moe and RNAi-mediated knockdown of Moesin resulted in clear disruption of mushroom body development (Table 1). Given that RNAi can have off target effects, we wished to determine whether the RNAi phenotypes were a specific result of a decrease in Moesin, therefore we reintroduced wild-type Moesin into both of the Moesin RNAi lines. Knockdown of Moesin resulted in an obvious deficit in α/β lobe development in an average of 85% of brains, which was reduced to 23% in brains of flies in which Moesin was co-expressed.

**Table 1:** Summary of mushroom body defects resulting from elav-GAL4 driven expression of UAS-Moe constructs

	Myc-Moe	Myc-MoeT559D	Moe-KD1	Moe-KD2	Myc-Moe; MoeKD1	Myc-Moe; MoeKD2
α/β lobes number^a^	104	70	84	88	84	74
Axon stalling^b^ (%)	21	4	10	5	12	11
Lobe missing^c^ (%)	35	89	67	70	5	5
Abnormal morphology^d^ (%)	2	7	10	11	5	8
Normal morphology (%)	52	0	14	16	79	76
γ lobes number	38	40	37	42	37	39
Thinner (%)	11	100	8	14	3	5
Normal morphology (%)	89	0	92	86	97	95

^a^The percentage of each lobe phenotype was calculated from the total number of brain hemispheres analyzed for each genotype (n). ^b^Brain hemispheres were scored as “axon stalling” when one or more partially elongated α/β lobes were present. ^c^The complete absence of one or more α/β lobes in a hemisphere was scored as “lobes missing”. ^d^Brain hemispheres presenting any other defects of lobe morphology were scored as “abnormal morphology”

Mushroom body defects ranged from misdirected or malformed lobes to the complete absence of α/β lobes, as well as axon arrest/stalling, in which the projection of α/β neurons from the peduncle is halted, resulting in partially formed lobes (Fig. 3). Additional defects in α/β lobe morphology included lobes that were thin or diminished, misdirected, misoriented, and those with defects in branching. Gamma lobe phenotypes were mild and observed as thinner lobes that were often distorted. Together these data demonstrate that wild-type levels of Moesin are required for normal axon outgrowth and guidance.

While generating the above-mentioned flies, we noticed that elav > Myc-MoeT559D resulted in photoreceptor deficits, displaying a rough eye phenotype, which is indicative of malformed or missing photoreceptor clusters, producing a rough, glassy look to the eye [37]. As *elav* is expressed in cells of neuronal progenitor origin including photoreceptors, the elav-GAL4 driver induces target gene expression in the eye as well as the brain [38]. The discovery of this eye development phenotype led to the examination of each of our transgenic Moesin expression lines by scanning electron microscopy to identify if altered Moesin expression resulted in visible disruption of photoreceptor development in the adult. SEM analysis revealed that expression of Myc-MoeT559D resulted in severe disorganization of bristles and ommatidia (Additional file 2: Figure S2A–E). We also observed that elav-GAL4 > Myc-MoeT559D flies lacked stereotypical climbing behavior, with all unable to climb and congregating at the bottom of vials (Additional file 2: Figure S2F), highlighting the importance of Moesin phosphoregulation in neurological function.

### Moesin regulates dendrite arborization and spine-like protrusion growth

**Fig. 4:**
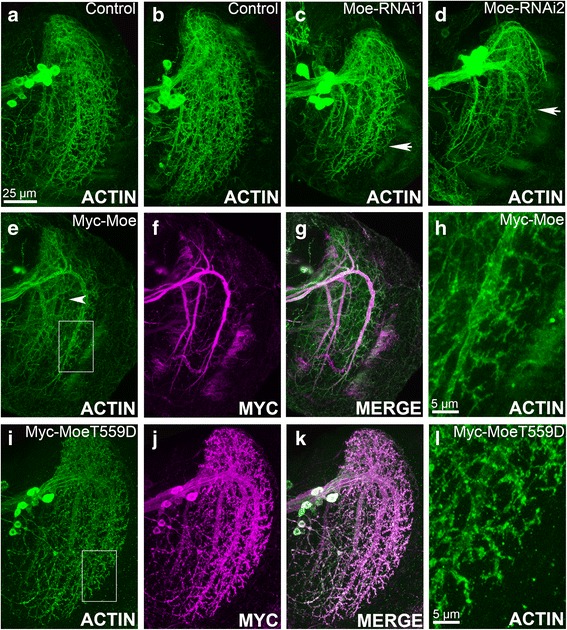
Altered Moesin expression disrupts dendritic arborization. Immunohistochemistry with anti-GFP (green) antibody on whole mount brains. All images are confocal projections through the optic lobe of the brain. **a**, **b**. 3A–GAL4 > Lifeact labels the dendritic arbor of the six neurons comprising the vertical system of LPTCs. **c**, **d**. Knockdown of *Moesin* results in defects in dendritic branching; arrows show stunted growth of VS branches. **e**–**g**. Overexpression of *Moesin* disrupts dendritic branching; arrow points to stunted VS branch. Anti-Myc staining (magenta) reveals that it localizes to the primary branches of the vertical system. **i**–**k**. Myc-MoeT559D distributes throughout the VS neurons, including the branchlets and spines. **l**. Expression of Myc-MoeT559D results in increased Lifeact staining the VS1 branch in comparison to expression of Myc-Moe (**h**)

In vertebrates, actin remodeling by Moesin has been shown to be crucial to dendritic spine growth and development [25, 26], therefore we next sought to interrogate whether Moesin is required for this process in the adult *Drosophila* central nervous system. The vertical system (VS) of lobula plate tangential cells (LPTCs), a group of visual system interneurons in the optic lobe, represent a model system particularly suited to the study of dendritic growth as these neurons display complex but stereotypical dendritic arborization [39]. In addition, dendrites in LPTCs have been shown to bear vertebrate spine-like protrusions that are actin-enriched [40]. To visualize dendritic morphology, the LPTC driver 3A–GAL4 was used to express Lifeact, which labels the dendrites of LPTCs with a particular concentration in the actin-rich dendritic protrusions. The characteristic arborization pattern of the six neurons, which form the VS of the LPTCs, is not altered by expression of Lifeact [40] (Fig. 4a, b). However, co-expression of Moesin RNAi with Lifeact revealed severely reduced dendritic projections (Fig. 4c, d, Table 2). Myc-Moe localized to primary branches and its expression also resulted in notable deficits in projections (Fig. 4e–g). Consistent with localization to actin-rich regions of the mushroom body, Myc-MoeT559D localized not only to the dendrite branches but also was strongly concentrated in branchlets and protrusions (Fig. 4i–k) with an apparent increase in the intensity of Lifeact, suggesting an increase in protrusion density (compare Fig. 4e to i and h to l). These data suggest that wild-type levels of Moesin are required for normal dendrite branching and arborization in *Drosophila* LPTCs, and activation of Moesin via phosphorylation may promote growth of dendritic spine-like protrusions.

**Table 2:** Summary of LPTC defects resulting from elav-GAL4 driven expression of UAS-Moe constructs

	Control	MoeKD	Myc-Moe	Myc-MoeT559D
Number^a^	19	21	15	18
Abnormal morphology^b^ (%)	0	76	40	22
Increased intensity of Lifeact in VS1 dendritic protrusions^c^ (%)	0	n/a^d^	0	44

^a^The percentage of each lobe phenotype was calculated from the total number of brain hemispheres analyzed for each genotype (n). ^b^Brain hemispheres were scored as “abnormal morphology” when one or more branches were missing, reduced in length or thinner than controls. ^c^The relative intensity of Lifeact in the dendritic protrusions was visually compared to that of the branch. If the protusion/branch intensity appeared higher than control brains, it was scored as “increased”. ^d^MoeKD was unable to be scored due to the severe branching defects. All analyses were performed by a blinded observer

### Moesin is required for long-term memory formation

**Fig. 5:**
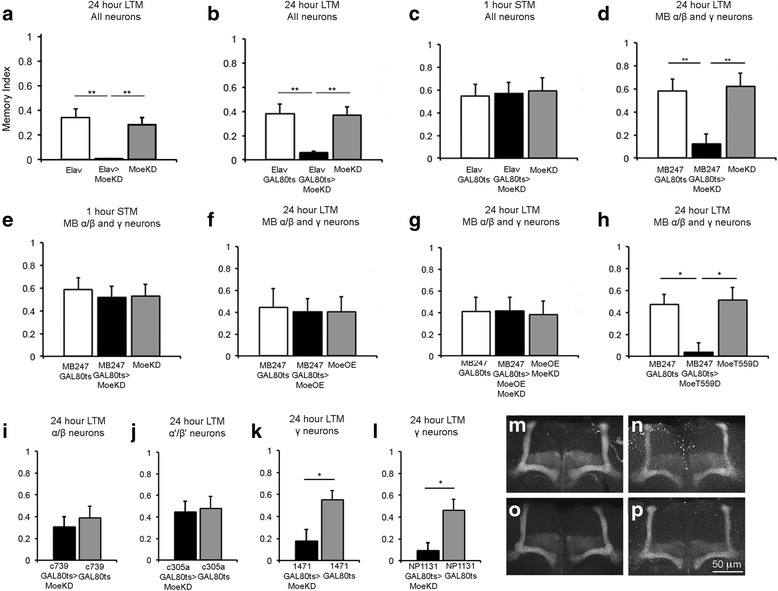
Moesin is required for long-term memory. **a**. 24-h long-term memory was significantly impaired by elav-GAL4 mediated knockdown of *Moesin* throughout development (ANOVA, post-hoc Tukey’s HSD, **p < 0.01). **b**. Knockdown of Moesin in adulthood with elav-Gal4 led to a significant impairment in 24-h long-term memory (ANOVA, post-hoc Tukey’s HSD, **p < 0.01). **c**. Short-term memory tested one hour following a one-hour training session was not significantly different. **d**. Knockdown of Moesin in the adult mushroom body with MB247-GAL4 resulted in a significant impairment in long-term memory (ANOVA, post-hoc Tukey’s HSD, **p < 0.01) **e**. One hour short-term memory was not affected by knockdown of Moesin in the adult mushroom body. **f**. No significant impairment in long-term memory resulted from MB247-driven expression of Myc-Moe in the adult mushroom body. **g**. Co-expression of Myc-Moe and Moe-KD1 with MB247 restored normal long-term memory. **h**. Expression of Myc-MoeT559D impaired long-term memory (ANOVA, post-hoc Tukey’s HSD, *p < 0.05). **i**. Knockdown of Moesin in α/β neurons had no significant effect on long-term memory **j**. Knockdown of Moesin in α’/β’ neurons had no significant effect on long-term memory. **k**. Knockdown of Moesin in γ neurons with 1471-GAL4 significantly impaired long-term memory (Student’s t-test, *p < 0.05). **l**. Knockdown of Moesin in γ neurons with NP1131-GAL4 also significantly impaired long-term memory (Student’s t-test, *p < 0.05). **m**–**p**. Anti FasII immunohistochemistry shows that no obvious developmental deficits are present in the mushroom bodies of flies that displayed long-term memory deficits. **m**. MB247 GAL80ts > MoeKD. **n**. MB247 GAL80ts > MoeT559D. **o**. 1471 GAL80ts > MoeKD. **p**. NP1131 GAL80ts > MoeKD

We next investigated the role of Moesin in memory formation in the repeat training courtship suppression assay [41–44]. In this assay, a male trained with a mated, unreceptive female will learn to recognize the rejection behavior of a mated female and therefore court less when presented with another mated female as compared to a male lacking this training. Pan-neuronal knockdown of Moesin had no significant impact on courtship of sham-trained Moesin knockdown males (Additional file 3: Figure S3), indicating that reduction of Moesin does not alter courtship behavior and any observed memory deficits would not be simply due to decreased courtship in this group. Pan-neuronal knockdown of Moesin resulted in a significant defect in 24-h long-term memory (Fig. 5a). As an intact mushroom body is critical for formation of courtship memory, the memory deficits observed were unsurprising. Therefore, in order to establish whether Moesin also plays a non-developmental role in long-term memory, the TARGET system was utilized to restrict Moesin knockdown to the adult brain. Flies were raised to adulthood at 19 °C (GAL4 repressed) then switched to 30 °C (GAL4 active) three days prior to testing to allow induction of RNAi expression. Tight induction of expression and negligible leakiness of the TARGET system was confirmed by western blotting (Additional file 4: Figure S4). Assessment of 24-h memory revealed a significant impairment in long-term memory, signifying that Moesin plays a non-developmental role in memory (Fig. 5b). We also assessed the integrity of short-term memory one hour after a one-hour training session, and found that knockdown of Moesin in the adult brain had no impact on short-term memory (Fig. 5c). As long-term courtship memory is mushroom body-dependent [34], we examined the specific requirement for Moesin in the mushroom body by restricting knockdown primarily to the α/β and γ neurons with the MB247-GAL4 driver [45]. This also resulted in impaired 24-h memory (Fig. 5d), and similarly short-term memory was unaffected (Fig. 5e). The requirement for Moesin in mushroom body neurons for normal long-term memory formation led us to investigate the effect of Moesin overexpression on long-term memory. MB247-driven expression of Myc-Moe in the adult mushroom body resulted in robust long-term memory, therefore elevated levels of Moesin had no significant effect on long-term courtship memory (Fig. 5f). In addition, co-expression of Myc-Moe rescued the long-term memory defect caused by knockdown of Moesin in the mushroom body (Fig. 5g), confirming that the memory deficit was specifically caused by a reduction in Moesin. While overexpression of wild-type Moesin had no impact, expression of constitutively active Moesin abolished long-term memory (Fig. 5h).

The three Kenyon cell types, α/β, α’/β’ and γ, differ in connectivity and have been previously shown to be functionally distinct with respect to their roles in long-term memory. We examined the spatial requirements for Moesin in the mushroom body by knocking down expression in each Kenyon cell subtype in the mature brain. The GAL4 drivers c739 and c305a drive expression in the α/β and α’/β’ Kenyon cell subtypes, respectively [46–48], and knockdown of Moesin with either of these drivers did not have a significant effect on long-term memory (Fig. 5i, j). Knockdown of Moesin in γ neurons with the 1471 and NP1131 drivers [49] recapitulated the impairment of long-term memory that was observed with elav and MB247 (Fig. 5k, l). Lastly, as an additional control, anti-FasII immunohistochemistry was performed on brains of flies of the genotypes that resulted in memory impairments to confirm normal mushroom body morphology (Fig. 5m). Taken together, these data indicate that Moesin expression is required in the γ neurons in order to form long-term courtship memory.

## Discussion

Here, we describe an essential role for Moesin in morphogenesis of mushroom body axons as well as a distinct non-developmental role in long-term memory. The examination of developmental deficits in the mushroom body of flies with reduced Moesin indicates an integral role for Moesin in axon projection, targeting, and branching. Increased Moesin expression also perturbed normal axonal outgrowth, but generally resulted in less severe developmental phenotypes, most likely because the majority of Moesin exists in an inactive conformation. However, the expression of the constitutively active Moesin resulted in complete disruption of mushroom body assembly, highlighting a critical role for phosphoregulation of Moesin in this developmental process.

Moesin was recently found to interact with the cell adhesion molecule Neuroglian, the sole *Drosophila* L1CAM [50]. Mutational analysis of Neuroglian identified a requirement for the ERM-interaction domain, to which Moesin binds, in the establishment of the mushroom body’s highly organized architecture [51]. *Neuroglian* mutants display severe mushroom body phenotypes including growth and guidance errors, missing lobes and branching defects [50, 51], similar to those herein that result from the modulation of Moesin expression. The deletion of the ERM interaction domain of Neuroglian, however, results in a phenotype in which aberrant axonal projections form a ball-like structure from continuous circular growth in the posterior of the brain [50, 51]. This phenotype has been described previously as axon stalling, however, it was subsequently characterized as a guidance error following the discovery that the defect results from a failure of axons to enter the peduncle [50, 51]. In contrast, our data reveal an axon stalling phenotype in which axon growth is arrested subsequent to branching from the peduncle and is observed in both *Moesin k*nockdown and overexpression brains. Additionally, the lack of any aberrant axonal accumulations in the posterior of brain hemispheres with missing mushroom body lobes indicates that these defects are likely the result of branching errors as axons either fail to bifurcate or subsequently segregate into vertical and medial lobes. The prevalence of this lobe formation defect in Neuroglian knockdown mushroom bodies and the importance of the ERM protein interaction domain to L1CAM-mediated axon branching in vertebrates suggests that reduction of Moesin expression may impair branching in part due to a reduced interaction with Neuroglian [50, 52]. The range of mushroom body defects resulting from the modulation of Moesin expression and activation highlights a central role for Moesin in axon growth and guidance.

Our data also reveal the importance of phosphoregulation of Moesin in photoreceptor and mushroom body development, as evidenced by the severe defects that result from the pan-neuronal expression of phosphomimetic Moesin. While the modulation of Moesin expression also led to serious defects in mushroom body assembly, no defect was observed in photoreceptors in which wild-type Moesin was knocked down or overexpressed. This finding is consistent with previous reports in which reduction of *Moesin* dosage by up to half did not impair photoreceptor development and only overexpression of Myc-MoeT559D led to guidance defects [53].

We also examined the role of Moesin in dendritic development and found that modulation of Moesin expression resulted in a disruption of the stereotypical dendritic arborization of the LPTC VS. Knockdown of Moesin resulted in a reduced dendritic field with fewer projections from multiple VS neurons, as did overexpression of wild-type Moesin. Constitutive activation of Moesin resulted in fewer branching deficits, but there was an increase in the density of dendritic protrusions on the VS1 branch**.** The complex phenotypes emerging from the modulation of Moesin suggest that it may be involved in multiple aspects of dendritic arborization. Previously characterized regulators of dendritic arborization in *Drosophila* include the Rho GTPases Rac1 and Rho1, which have opposing effects on the growth and complexity of dendrites. Rac1 promotes dendritic branching and extension while Rho1 restricts both branching and branch length [54–56]. Moesin has been shown to negatively regulate Rho1 activity in *Drosophila* epithelial cells [57, 58] and neurons [59], therefore, the lack of dendritic projections in some Moesin-knockdown neurons may be the result of Rho1 hyperactivity. *Rho1* null MB neurons display increased dendritic volume, whereas constitutively active Rho1 results in reduced dendritic volume in the mushroom body [55], which is consistent with the hypothesis that Moesin negatively regulates Rho1. However, the regulatory interaction may be more complex, as Rho1 has also been demonstrated to act upstream of Moesin [11]. In addition, expression of constitutively active Moesin and overexpression of wild-type Rac1 in LPTCs [40] both result in an increased number of dendritic protrusions, suggesting that Moesin may interact with both Rac1 and Rho1 to regulate growth of dendritic protrusions.

While we found that expression of constitutively active Moesin resulted in severely reduced and malformed mushroom body axons, most dendrites displayed arbors with typical field coverage. The molecular pathways for axon and dendritic morphogenesis are distinct, and insight into some of the molecular mechanisms that are responsible have been provided by Lee and colleagues who demonstrated that Rho GTPases play contrasting molecular roles in axon and dendrite morphogenesis [55]. For example, although they display altered dendritic development, *Rho1* null flies develop normal mushroom body axons. *Rac1* mutants, on the other hand, display severe defects in mushroom body axon growth and guidance but display far milder phenotypes in mushroom body dendrites [60] and do not disrupt dendritic branching in LPTCs [40]. Interestingly, the defects in mushroom body axon morphogenesis and the increase in dendritic protrusions in the LPTC visual system that we observed on expression of constitutively active Moesin are both similar phenotypes to those that result from increased Rac1 activity [40, 55]. *Rac1* and *Neuroglian* also interact genetically in mushroom body axons, with a *Rac1* mutant exacerbating the growth and guidance deficits resulting from both loss and gain of function of *Neuroglian,* suggesting *Rac1* may act both up and downstream [51]. Assays for Rac1 and Rho1 activity in presence of WT and mutant forms of Moesin (i.e. knockdown, and expression of T559A and T559D), in combination with analysis of Moesin phosphorylation in the presence of *Rac1* and *Rho1* mutants will be valuable in determining whether Moesin acts upstream and/or downstream of Rho GTPases.

We also provide evidence for an adult-specific role of Moesin in long-term memory formation. We found that pan-neuronal knockdown of Moesin throughout development had no effect on courtship activity, with males displaying the full repertoire of courtship behaviors and no difference in the amount of time spent in courtship behavior between sham control and Moesin knockdown flies. Thus wild-type levels of Moesin are not required in the brain for normal courtship activity. Long-term courtship memory was impaired, as would be expected since formation of this memory is dependent on an intact mushroom body [34]. However, conditional knockdown of Moesin in all neurons of the adult brain led to similar defects in long-term memory. This argues strongly for a post-developmental role for Moesin in long-term memory, which is not attributable to a non-specific disruption of cellular function or role in general neurotransmission, as one-hour short-term memory, which is also dependent on an intact mushroom body [34] was not affected. By targeting Moesin knockdown in the adult specifically to the neurons that comprise the mushroom body, the requirement for Moesin in long-term memory was traced to the γ neurons of the mushroom body. While the precise molecular mechanisms behind courtship learning are still largely unresolved, several steps in the acquisition and consolidation of memory have been elucidated. Loss of the cytoplasmic polyadenylation element–binding protein Orb2 results in a specific impairment in long-term memory formation, and restoration of Orb2 in the γ neurons during or immediately after a training session is sufficient to rescue this long-term memory deficit [44]. The activation of Orb2 requires input to the mushroom body from aSP13 dopaminergic neurons during both acquisition and consolidation, which is dependent on the presence of the dopamine receptor DopR1 in γ neurons [61]. During consolidation, this activation results in the formation of a complex between the two Orb2 isoforms, Orb2A and Orb2B at synapses. This Orb2 complex then induces translation of CaMKII [61], a protein critical for persistence of memory [62]. Transcriptional modulators have also been found to act in the γ neurons to facilitate normal long-term memory. Overexpression of the histone deacetylase HDAC4 specifically in the γ neurons of adult flies impairs long-term memory [42], as does knockdown of *Rpd3 (HDAC1)* [43]. Together these data are consistent with the synapses of mushroom body γ neurons being a likely site of the protein synthesis-dependent plastic modifications that underpin long-term courtship memory in *Drosophila* **.** These plastic changes at synapses are highly contingent upon actin remodeling within particular compartments to enable the dynamic structural modifications in neuronal morphology [13, 15, 16]. As a key regulator of the actin cytoskeleton in neurons, we hypothesize that training results in activation of Moesin, which promotes actin rearrangements that underpin the morphological changes at specific synapses. This is consistent with the lack of an effect from the overexpression of wild-type Moesin, which was largely cytoplasmic and inactive.

## Conclusions

In summary, we provide evidence that the actin-binding protein Moesin is necessary for both normal development of the mushroom body as well as a mushroom body-dependent post-developmental role in long-term memory. These data, taken together with the evidence that Moesin regulates cytoskeletal rearrangement and promotes the growth of dendritic spine-like protrusions in *Drosophila* and spine growth in mammals [25, 26], suggest that Moesin may be a key facilitator of the morphological changes in neurons that occur during long-term memory consolidation.

## Methods

### Fly strains

All flies were cultured on standard medium on a 12-h light/dark cycle and maintained at a temperature of 25 °C unless otherwise indicated. Canton S flies were used as wild-type controls. *P{w[+mW.hs] = GawB}elav[C155]* (elav-GAL4, #458); *w[1118];P{w[+mC] = UASMoe.IR.327–775}3* (MoeKD2, #8629); *w[1118];P{w[+mC] = UASMoe.MYC.K}2 (*Myc-Moe, #8631); *w[1118];P{w[+mC] = UASMoeT559D.MYC}2* (Myc-MoeT559D, #8630); y [1] w[67c23]; P{w[+mW.hs] = GawB}Hr39[c739], (c739-GAL4, #7362); *w[1118];P{w + mW.hs = GawB}c305a* (c305a-GAL4); *w[1118];P{w + mW.hs = GawB}1471* (1471-GAL4, #9465); *y* [1] w[*]; *P{w[+mW.hs] = GawB}3A* (3A–GAL4, #51629) and *y* [1] w[*]; P{y[+t*] w[+mC] = UAS-Lifeact-GFP}VIE-260B (Lifeact, #35544) were obtained from the Bloomington *Drosophila* Stock Center, stock numbers indicated in brackets. *w* ^*^ *; P{w + mC = tubP-GAL80ts}10* (tubP-GAL80^ts^), w[*];P{w[+m*] = Mef2-GAL4.247}3 [63] (MB247-GAL4) and *w(CS10)* strains were kindly provided by R. Davis (The Scripps Research Institute, Jupiter, FL). *w[1118]; P{w[+mC] (UASMoe IR.528–897}2* (MoeKD1, Transformant ID110654) was obtained from the Vienna Drosophila Resource Center. All strains used for behavioral testing and analysis of brain development were outcrossed for a minimum of five generations to *w(CS10)* flies. Homozygous lines harbouring *w(CS10); P{w + mC = tubP-GAL80ts}10* and the appropriate GAL4 drivers were generated by standard genetic crosses.

### Immunohistochemistry

Whole flies were fixed in PFAT/DMSO (4% paraformaldehyde in 1X phosphate buffered saline + 5% dimethyl sulfoxide + 0.1% Triton X-100) for one hour then washed in PBT (1Xphosphate buffered saline + 0.5% Triton X-100). Brains were microdissected in PBT then post fixed in PFAT/DMSO for 20 min and stored in methanol at −20 °C. Following rehydration in PBT, brains were blocked in immunobuffer (5% normal goat serum in PBT) for >2 h at room temperature. They were then incubated overnight at room temperature with primary antibody and subsequently incubated overnight at 4 °C with secondary antibody (goat anti-mouse Alexa488, goat anti-mouse Alexa555, goat anti-rabbit Alexa488, or goat anti-rabbit Alexa555, Molecular Probes, 1:200) and mounted with Antifade mounting medium (4% n-propyl gallate in 90% glycerol + 10% phosphate buffered saline). The following antibodies were used: Anti-Moesin (1:5000) kind gift from D. Kiehart [6]; anti-Myc (1:50) developed by J. M. Bishop and anti-ELAV 9F89A clone (1:100) developed by G.M. Rubin, both of which were obtained from the Developmental Studies Hybridoma Bank developed under the auspices of the NICHD and maintained by The University of Iowa, Department of Biology, Iowa City, IA. For confocal microscopy, optical sections were taken with a Leica TCS SP5 DM6000B Confocal Microscope. Image stacks taken at intervals of 1 μm (whole brain) or 0.5 μm (MB and LPTCs) and were processed with Leica Application Suite Advanced Fluorescence (LAS AF) and ImageJ software.

### Western blot

Flies were collected in tubes and frozen in a dry ice/ethanol bath. The tubes were vortexed to snap the heads from the bodies, and the heads were collected. Cytoplasmic extracts were prepared by homogenizing heads with a disposable mortar and pestle in RIPA buffer (150 mM sodium chloride, 1% Triton X-100, 1% sodium deoxycholate, 0.1% sodium dodecyl sulfate, 25 mM Tris, pH 8.0). Following centrifugation at 13,000 g for 2 min at 4 °C, the supernatant was retained as the cytoplasmic fraction. Protein concentration was then determined with the Pierce BCA Protein Assay Kit (ThermoFisher Scientific). 20 μg of each sample was loaded onto a 10% sodium dodecyl sulfate-polyacrylamide gel electrophoresis gel and resolved at 200 V. Protein was transferred onto nitrocellulose and blocked for >2 h in 5% skim milk powder in TBST (50 mM Tris, 150 mM NaCl, 0.05% Tween-20, pH 7.6). The membrane was incubated overnight at 4 °C in primary antibody and one hour in secondary antibody. Antibodies used were anti-Moesin (D. Kiehart, Duke University, 1:50,000), anti-Myc (1:100) and anti α-tubulin (12G10 clone, developed by J. Frankel and *M. nelson*, Developmental Studies Hybridoma Bank, 1:500). Detection was performed with ECL Plus (GE).

### Behavioral analyses

The repeat training courtship suppression assay [37, 41–44] was used to assess one-hour and 24-h memory. In this assay, a male trained with a mated, unreceptive female will learn the rejection behavior of a mated female and therefore court less when presented with a mated female in the future as compared to an untrained male. All behavioral assays and statistical analyses were performed as previously described [43]. A training session consists of pairing a virgin male with a female who was mated the previous night for 1 to 7 h. The male is left to court the mated female for the duration of the training session, after which time the female was removed. A one-hour training session was administered for the analysis of short-term memory, while a seven-hour training session was applied in long-term memory assessment. In parallel, a naïve “sham” male of the same genotype was housed alone. Long-term memory was measured 24 h after training by pairing each male with another freshly mated female and scoring his courtship activity (licking, chasing, or orienting toward the female, wing extension and vibration) over a ten-minute period. Short-term memory was assessed in the same manner one hour after the training session. In order to generate a memory score from this courtship data a memory index was calculated by comparing the percentage of the ten-minute period spent engaging in courtship behavior (courtship index) against the mean of the sham flies of its genotype (n≥16/group). Memory was measured on a scale of 0 to 1, with 1 being the highest memory score possible, and a score of 0 indicating memory is no different than untrained sham controls.

## Additional files

Additional file 1: Figure S1. Confocal projections of brains expressing Lifeact and counterstained with the neuropil marker nc82. A-*C. frontal* confocal projection showing localization of Lifeact (green) primarily to the mushroom body lobes and glomeruli. D-F. Posterior confocal projection showing localisation of Lifeact to the optic lobes and calyx of the mushroom body. Abbreviations: MB, mushroom body lobes; G, glomeruli; C, calyx; OL, optic lobe. (PDF 4815 kb)

Additional file 2: Figure S2. Eye and locomotor phenotypes resulting from elav-GAL4-driven knockdown and overexpression of Moesin. A-E. Scanning electron micrographs of the Drosophila eye. A. elav/+ control. B elav > MoeKD1. C. elav > MoeKD2. D. elav > Myc-Moe. E. elav > Myc-MoeT559D. F. Left vial, elav/+ control. Right vial, elav > Myc-Moe. (PDF 2264 kb)

Additional file 3: Figure S3. Courtship activity of sham trained flies from each of the courtship suppression assays. Sham controls were exposed to the same training procedure as the trained flies but were not exposed to a female. The lack of significant difference in courtship activity between the genotypes indicates that courtship activity itself was not affected by genetic manipulation of Moesin. (PDF 1007 kb)

Additional file 4: Figure S4. Temperature sensitive regulation of Moesin RNAi and transgene expression. A. Whole cell lysates were prepared from heads from *elav-GAL4/+; tub-GAL80ts/+* and *elav-GAL4/+; tub-GAL80ts/UAS-MoeRNAi* flies that were raised and maintained at 19°C, or raised at 19°C then switched to 30°C for three days prior to harvest. The blot was probed with anti-Moesin and anti-α-tubulin antibody was used as a loading control. B. Whole cell lysates were prepared from heads from *elav-GAL4/+; tub-GAL80ts/+* and *elav-GAL4/+; tub-GAL80ts/UAS-Myc-Moe* and *elav-GAL4/+; tub-GAL80ts/UAS-Myc-MoeT559D* flies that were raised and maintained at 19°C, or raised at 19°C then switched to 30°C for three days prior to harvest. The blot was probed with anti-Myc and anti-α-tubulin antibody was used as a loading control. (PDF 849 kb)
