# Supplementary figures and images for: The ERM protein Moesin is essential for neuronal morphogenesis and long-term memory in Drosophila
Source: Mol Brain. 2017 Aug 29;10:41. doi: 10.1186/s13041-017-0322-y (PMC5576258; doi:10.1186/s13041-017-0322-y)

**Figure S1**

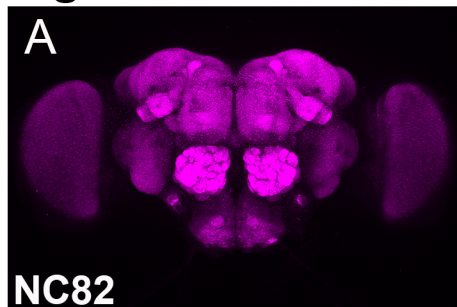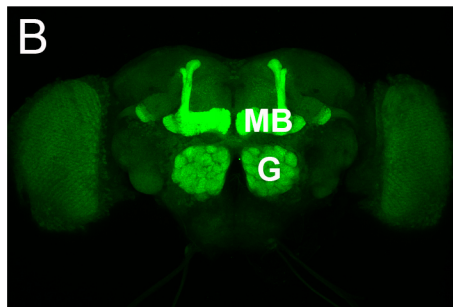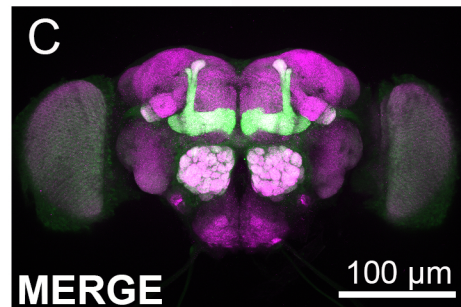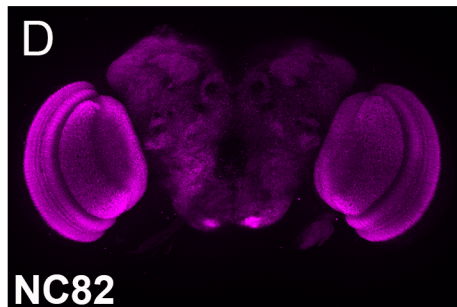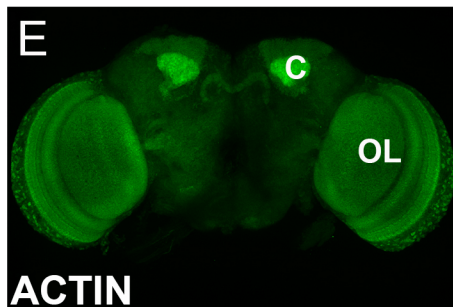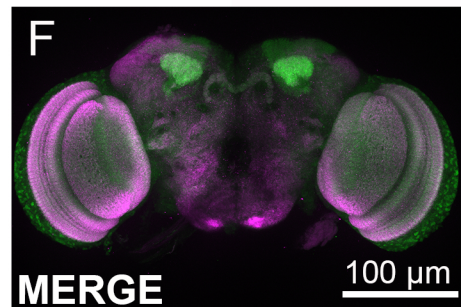

Supplement: Supplementary file 1 — Confocal projections of brains expressing Lifeact and counterstained with the neuropil marker nc82. A-C. frontal confocal projection showing localization of Lifeact (green) primarily to the mushroom body lobes and glomeruli. D-F. Posterior confocal projection showing localisation of Lifeact to the optic lobes and calyx of the mushroom body. Abbreviations: MB, mushroom body lobes; G, glomeruli; C, calyx; OL, optic lobe. (PDF 4815 kb) [file 13041_2017_322_MOESM1_ESM.pdf]

# Figure S2

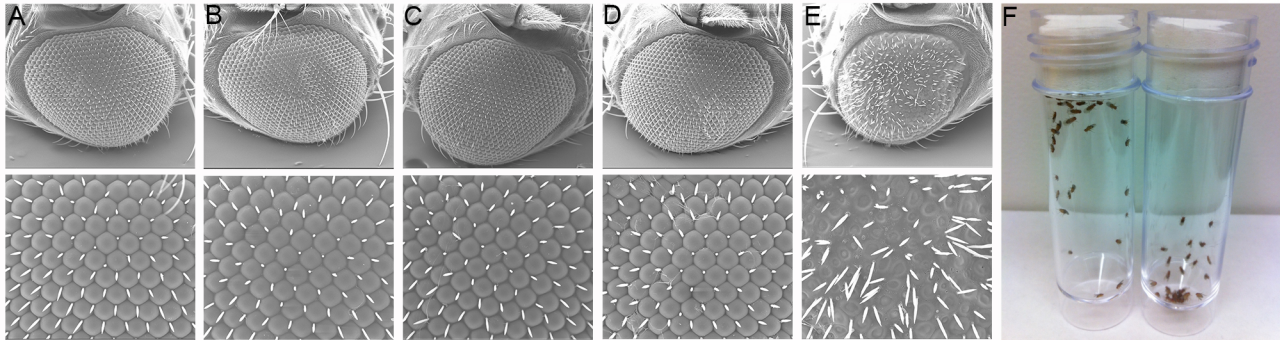

Supplement: Supplementary file 2 — Eye and locomotor phenotypes resulting from elav-GAL4-driven knockdown and overexpression of Moesin. A-E. Scanning electron micrographs of the Drosophila eye. A. elav/+ control. B elav > MoeKD1. C. elav > MoeKD2. D. elav > Myc-Moe. E. elav > Myc-MoeT559D. F. Left vial, elav/+ control. Right vial, elav > Myc-Moe. (PDF 2264 kb) [file 13041_2017_322_MOESM2_ESM.pdf]

# Figure S3

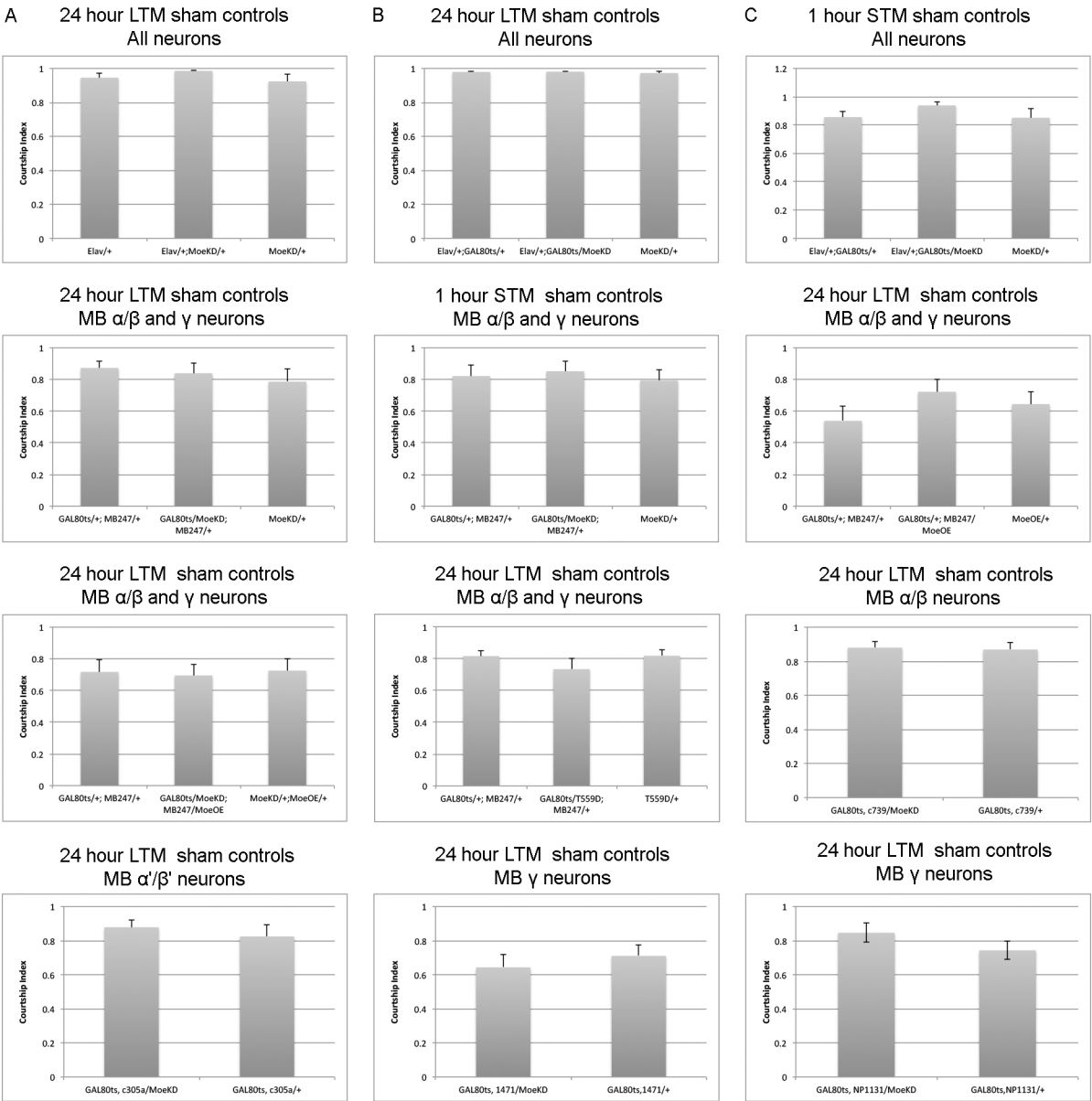

Supplement: Supplementary file 3 — Courtship activity of sham trained flies from each of the courtship suppression assays. Sham controls were exposed to the same training procedure as the trained flies but were not exposed to a female. The lack of significant difference in courtship activity between the genotypes indicates that courtship activity itself was not affected by genetic manipulation of Moesin. (PDF 1007 kb) [file 13041_2017_322_MOESM3_ESM.pdf]
