# Supplementary material for: The ERM protein Moesin is essential for neuronal morphogenesis and long-term memory in Drosophila
Source: Mol Brain. 2017 Aug 29;10:41. doi: 10.1186/s13041-017-0322-y (PMC5576258; doi:10.1186/s13041-017-0322-y)
Supplement: Supplementary file 4 — Temperature sensitive regulation of Moesin RNAi and transgene expression. A. Whole cell lysates were prepared from heads from elav-GAL4/+; tub-GAL80ts/+ and elav-GAL4/+; tub-GAL80ts/UAS-MoeRNAi flies that were raised and maintained at 19°C, or raised at 19°C then switched to 30°C for three days prior to harvest. The blot was probed with anti-Moesin and anti-α-tubulin antibody was used as a loading control. B. Whole cell lysates were prepared from heads from elav-GAL4/+; tub-GAL80ts/+ and elav-GAL4/+; tub-GAL80ts/UAS-Myc-Moe and elav-GAL4/+; tub-GAL80ts/UAS-Myc-MoeT559D flies that were raised and maintained at 19°C, or raised at 19°C then switched to 30°C for three days prior to harvest. The blot was probed with anti-Myc and anti-α-tubulin antibody was used as a loading control. (PDF 849 kb) [file 13041_2017_322_MOESM4_ESM.pdf]

Figure S4

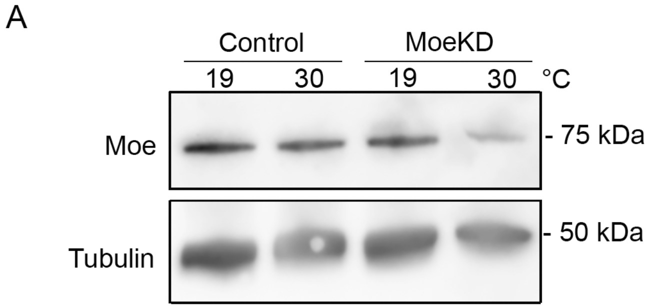
A
Control
MoeKD
19
30
19
30
°C
Moe
- 75 kDa
Tubulin
- 50 kDa

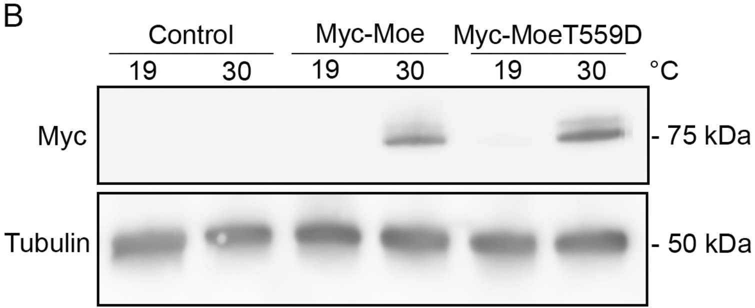
B
Control
Myc-Moe
Myc-MoeT559D
19
30
19
30
19
30
°C
Myc
- 75 kDa
Tubulin
- 50 kDa
